# Selective Tissue Distribution Mediates Tissue-Dependent PPARγ Activation and Insulin Sensitization by INT131, a Selective PPARγ Modulator

**DOI:** 10.3389/fphar.2017.00317

**Published:** 2017-05-30

**Authors:** Xinni Xie, Wei Chen, Ning Zhang, Mei Yuan, Cheng Xu, Zhibing Zheng, Hua Li, Lili Wang

**Affiliations:** ^1^Beijing Institute of Pharmacology and ToxicologyBeijing, China; ^2^State Key Laboratory of Toxicology and Medical CountermeasuresBeijing, China; ^3^State Key Laboratory of Environmental Chemistry and Ecotoxicology, Research Center for Eco-Environmental Sciences, Chinese Academy of SciencesBeijing, China; ^4^School of Pharmaceutical Engineering, Life Science and Biology Pharmacy College, Shenyang Pharmaceutical UniversityShenyang, China

**Keywords:** PPARγ, INT131, tissue distribution, selective PPARγ modulator, insulin sensitivity

## Abstract

The mechanisms underlying the enhancement of insulin sensitivity by selective peroxisome proliferator-activated receptor γ modulators (sPPARγMs) are still not completely known. Here, the representative sPPARγM, INT131, was used as a probe to investigate the insulin-sensitizing mechanisms of sPPARγM in the context of tissue selective compound distribution and PPARγ regulation. First, 30 mg kg^−1^ INT131 was observed to produce an insulin-sensitizing effect comparable to that of 10 mg kg^−1^ rosiglitazone (RSG) in both db/db and DIO mice using the oral glucose and insulin tolerance tests. Similar to RSG, INT131 significantly increased brown adipose tissue (BAT) mass and adipocyte size and up-regulated the expression of BAT-specific genes. Compared with RSG, INT131 exhibited greater potency in inducing white adipose tissue (WAT) browning, decreasing adipocyte size, and increasing BAT-specific and function-related gene expression in subcutaneous WAT (sWAT). However, it did not induce hepatomegaly or hepatic steatosis, which is associated with lower levels of lipogenic genes expression. Pharmacokinetic analysis reveals that in contrast with RSG, INT131 shows higher Cmax, and much longer residency time (AUC_0−12h_), as well relatively lower elimination rate in adipose tissues and skeletal muscle, this demonstrated INT131 distributed predominantly in adipose tissue. Whereas, INT131 was less abundant in the liver. These results thus suggest that the tissue-selective distribution underlies INT131's selective PPARγ modulation. Compounds favoring adipose tissue may aid in development of better, safer sPPARγM to address the insulin resistance of diabetes.

## Introduction

Insulin resistance (IR) is a root cause of type 2 diabetes mellitus (T2DM) and represents an efficient therapeutic target (Colca, 2015). Nuclear receptor peroxisome proliferator-activated receptor γ (PPARγ), a ligand-dependent transcription factor, ameliorates IR by controlling the expression of genes responsible for adipocyte differentiation, lipid metabolism, glucose homeostasis, and inflammation in insulin target tissues (Lehrke and Lazar, 2005; Ahmadian et al., 2013). The classical thiazolidinedione (TZD) group of PPARγ full agonists, including rosiglitazone (RSG) and pioglitazone, consists of IR-targeting agents (Yki-Jarvinen, 2004). However, widespread prescription of TZDs is greatly limited by severe side effects, including weight gain, oedema (Guan et al., 2005), an increased risk of congestive heart failure (Nissen and Wolski, 2007), and bone fracture (Wei and Wan, 2011).

Owing to the successful development of a selective estrogen receptor modulator demonstrating tissue-selective effects and differential gene modulation profiles (Shang and Brown, 2002) and increased understanding of the molecular mechanisms underlying PPARγ regulation, selective PPARγ modulators (sPPARγMs), a class of PPARγ ligands that confer beneficial insulin-sensitizing effects with fewer side effects than full PPARγ agonists, are considered desirable targets for development of a new generation of safer insulin sensitizers (Higgins and Depaoli, 2010). A distinct binding mode between a ligand and the ligand-binding domain of PPARγ receptor could lead to differential transcriptional cofactor recruitment to or displacement from this receptor. The quantities or types of cofactors present in different cells also affect this process, ultimately leading to tissue- or cell-specific gene expression. The binding mode and cellular context underlie the molecular mechanisms of sPPARγM. However, investigation of cofactor recruitment/dissociation has been limited by variations in the types of PPARγ cofactors present and their abundances in different tissues, and almost all previous studies have been based on *in vitro* examinations with only parts of cofactors. In 2010, Choi et al. found that cyclin-dependent kinase 5 (CDK5)-mediated phosphorylation at serine 273 of PPARγ (pSer273PPARγ) is a critical link between obesity and insulin resistance. pSer273PPARγ inhibition selectively induces expression of specific subsets of PPARγ target genes that are responsible for insulin sensitization instead of adipogenic related genes, and thus constitutes another potential mechanism underlying the anti-diabetic effects of sPPARγMs (Choi et al., 2010), although this has not been systematically demonstrated (Malapaka et al., 2012; Weidner et al., 2013). Additionally, the physiochemical property of a given compound will also greatly affect its characteristics of pharmacokinetics and ultimately the overall pharmacodymics, and even will determine the fate of it. Thus, sPPARγM-mediated tissue-selective PPARγ activation and gene expression related to selective PPARγ regulation in ameliorating IR remain to be elucidated.

The specific ligand of a receptor is crucial for determining the target function of the receptor and related regulatory mechanisms, and particularly for thoroughly understanding the characteristics of receptor-selective modulation. A phase IIb clinical trial has been completed for INT131, a promising non-TZD sPPARγM that improves hyperglycaemia in both animal diabetes models and T2DM patients, with effects comparable to those of TZDs but with fewer side effects than full PPARγ agonists (Motani et al., 2009; Dunn et al., 2011; Lee et al., 2012; DePaoli et al., 2014). INT131 binds to the ligand-binding pocket of PPARγ receptor, partly overlapping with the TZD binding site, but does not directly contact with Tyr 473 in helix H12, which is the binding site associated with full PPARγ agonism (Bruning et al., 2007; Einstein et al., 2008; Motani et al., 2009). In accordance with its unique structure and interaction mode with PPARγ, INT131 recruits or disassociates from transcriptional coactivators or co-repressors in a manner distinct from that of RSG (Motani et al., 2009).

The concentration of drug in target tissue is a key determinant of its efficacy, whereas the concentration in non-target tissue constitutes a major determinant of the side effects. Given the selective pharmacology of sPPARγMs, the relationship between their tissue-selective effects and differential tissue distribution has not previously been addressed. Thus, in this study, INT131 was used as a probe to investigate the tissue-selective effects of sPPARγM and elucidate the mechanisms underlying the insulin-sensitizing activity of INT131 compared with that of RSG. Moreover, we sought to determine whether these differential effects/mechanisms are related to specific drug pharmacokinetics, including tissue distribution and concentrations in primary target tissues (liver, adipose tissue, and skeletal muscle).

## Materials and methods

### Chemical compounds

The compounds RSG (MW: 379.4) and INT131 (MW: 514) were synthesized by the new drug design center at our institute. Purity (>99%) and structure were confirmed through high performance liquid chromatography (HPLC), mass spectrometry and H-nuclear magnetic resonance. The compound structures are shown in (Supplementary Figure 5).

### Animal studies

All animal experiments were performed according to procedures approved by the Animal Experimentation Ethics Committee of the Beijing Institute of Pharmacology and Toxicology for animal care, handling and termination. Eight-week-old male homozygous C57BLKS/J db/db mice were obtained from the Model Animal Research Centre of Nanjing University (Nanjing, China) and fed a standard mouse diet. Male C57BL/6 mice (4 weeks old) were obtained from the Laboratory Animal Centre of the Beijing Institute of Pharmacology and Toxicology and fed a high-fat, high-sugar diet for 16 weeks to induce diet-induced obesity (DIO). Animals were maintained under temperature-, humidity-, and light-controlled conditions (22°C, 50% humidity, 12-h day/night schedule), and mice had *ad libitum* access to water. Mice were treated by gavage once daily with RSG (10 mg kg^−1^), INT131 (10 or 30 mg kg^−1^), or vehicle for 3 weeks. Compounds were dissolved in DMSO and suspended in 0.5% carboxymethylcellulose. Body weight (BW) and fasting blood glucose were recorded regularly during the treatment period. After 14 d of treatment, db/db mice were subjected to insulin tolerance tests (ITT) and fasted for 6 h prior to the intraperitoneal (i.p.) injection of 1 U kg^−1^ insulin, and blood glucose was estimated at the indicated time points. After 14 days of treatment, DIO mice were fasted for 12 h prior to oral glucose tolerance test (OGTT). Blood glucose was monitored at 0, 30, 60, 90, and 120 min after the oral administration of 1 g kg^−1^ dextrose. At the end of the experimental period, mice were fasted for 12 h, and blood samples were taken from the orbital vein to assess serum biochemical parameters. Interscapular brown adipose tissue (iBAT), epididymal white adipose tissue (eWAT), subcutaneous inguinal white adipose tissue (sWAT), liver and skeletal muscle were excised, weighed and rapidly frozen in liquid nitrogen for subsequent gene expression and western blot analysis.

### Metabolic parameters

Serum triglyceride (TG), cholesterol, free fatty acid (FFA), and glucose levels were measured by using commercial kits (Nanjing Jiancheng Bioengineering Institute, Nanjing, China) according to the manufacturer's instructions. Serum adiponectin, monocyte chemotactic protein 1(MCP1), Interleukin-6 (IL-6), Receptor activator of nuclear factor kappa-B ligand (RANKL), and Adrenocorticotropic Hormone (ACTH) were quantified using a multiplex Luminex assay (Millipore/Linco Research).

### Lipids level in the liver

To determine TG and total cholesterol levels in the liver, 50 mg of frozen tissue was homogenized with a TissueLyserII (Qiagen, Germantown, MD, USA) in 1 ml of chloroform/methanol (2:1, v/v), after which 200 μL of 0.05 M KCl was added, and the solution was centrifuged at 5,000 rpm for 10 min. The aqueous phase was removed, and the organic phase was filtered and dried by nitrogen flushing. After evaporation of the chloroform under nitrogen, lipid samples were resuspended in isopropyl alcohol, and TG and total cholesterol content were determined with enzymatic assays.

### Immunohistochemistry and determination of adipocyte cell size

sWAT, iBAT, and liver samples were fixed, embedded in paraffin and sectioned. Sections were cut at a thickness of 5 μM and stained with haematoxylin/eosin (HE). Liver sections as well as adipose sections from at least 3 mice per group were also stained with HE. Adipocyte (*n* = 100 adipocytes) diameter was measured using Image J software, and the average diameter was recorded for each animal.

### RNA extraction, cDNA synthesis, and real-time PCR

Total RNA was isolated from tissues with a High-Purity Total RNA Extraction Kit (BioTeke Corporation, Beijing, China) according to the manufacturer's instructions. RNA was reverse-transcribed into cDNA with a High Capacity cDNA Reverse Transcription Kit (Transgenes Corporation, Beijing, China). Real-time PCR was carried out on an ABI 7300 Real-time PCR System (Applied Biosystems, Foster City, CA, USA) using SYBR Green PCR Master Mix (Roche Molecular Biochemicals, Mannheim, Germany). Relative gene expression levels were normalized to the β-actin gene and quantified by using the 2^−ΔΔCt^ method. Primer sequences are listed in (Supplementary Table 1).

### Western blotting

To obtain tissue lysates, eWAT and sWAT were homogenized in 10% sodium dodecyl sulfate buffer containing protease and phosphatase inhibitors. An antibody targeting PR domain containing 16 (PRDM16) (Abcam) was used for western blotting. Bands were detected with ECL enhanced chemiluminescence detection reagents (Applygen, Beijing, China) and scanned with an Alpha Imager 5500 (Alpha Innotech, San Leandro, CA, USA) imaging densitometer.

### RSG and INT131 concentrations in plasma and tissue

DIO mice (*n* = 12) were subjected to a single dose of vehicle, RSG (10 mg kg^−1^) or INT131 (30 mg kg^−1^) by oral gavage, and then plasma, liver, skeletal muscle, iBAT, sWAT, and eWAT were collected at 0.5, 4, and 12 h after administration (*n* = 3 at each time point) and rapidly stored at −80°C for further assays. RSG and INT131 concentrations in plasma and tissue were quantitatively analyzed using an Agilent 1290 Infinity UHPLC system coupled with an Agilent 6410B triple quadrupole mass spectrometer (Agilent Technologies, Santa Clara, CA). RSG, INT131 and propranolol (internal standard) were eluted from a CAPCELL PAK MGII C18 column (dp = 3 μM, 2.0 × 100 mm, Shiseido, Japan) using a mobile phase gradient (A: water with 0.1% formic acid and 5 mM ammonium formate, B: acetonitrile with 0.1% formic acid). The following RSG gradient was run: 0–1 min hold at 30% B; 1–1.8 min, linear gradient to 75% B; hold at 75% B for 1.5 min. The following INT131 gradient was run: 0–0.6 min hold at 40% B; 0.6–1 min, linear gradient to 75% B; 1–1.2 min, linear gradient to 98% B; hold at 98% B for 2 min. The flow rate was 0.35 ml/min. Analytes were detected in positive ion mode via multiple reaction monitoring: RSG, 358.2–135.1 m/z, INT131, 515.0–129.1, propranolol, 260.0–116.2 m/z. The lower limits of detection were 50 ng Ml^−1^ for RSG and INT131 in plasma and 1 ng g^−1^ for tissue samples. The recovery was >92% with this method. The inter-day and intra-day precisions (RSD) were <10% and <7%, respectively.

### Statistical analyses

For two-group comparisons, statistical significance was determined with unpaired two-tailed Student's *t*-test unless otherwise stated. For multiple comparisons, statistical analysis consisted of one-way ANOVA followed by Tukey's multiple comparisons test calculated in GraphPad Prism 5.0 software. *P* < 0.05 was considered to be statistically significant.

## Results

### INT131 exhibits different tissue-selective effects compared with those of RSG but equivalent insulin-sensitizing activity in both db/db and DIO mice

**Figure 1:**
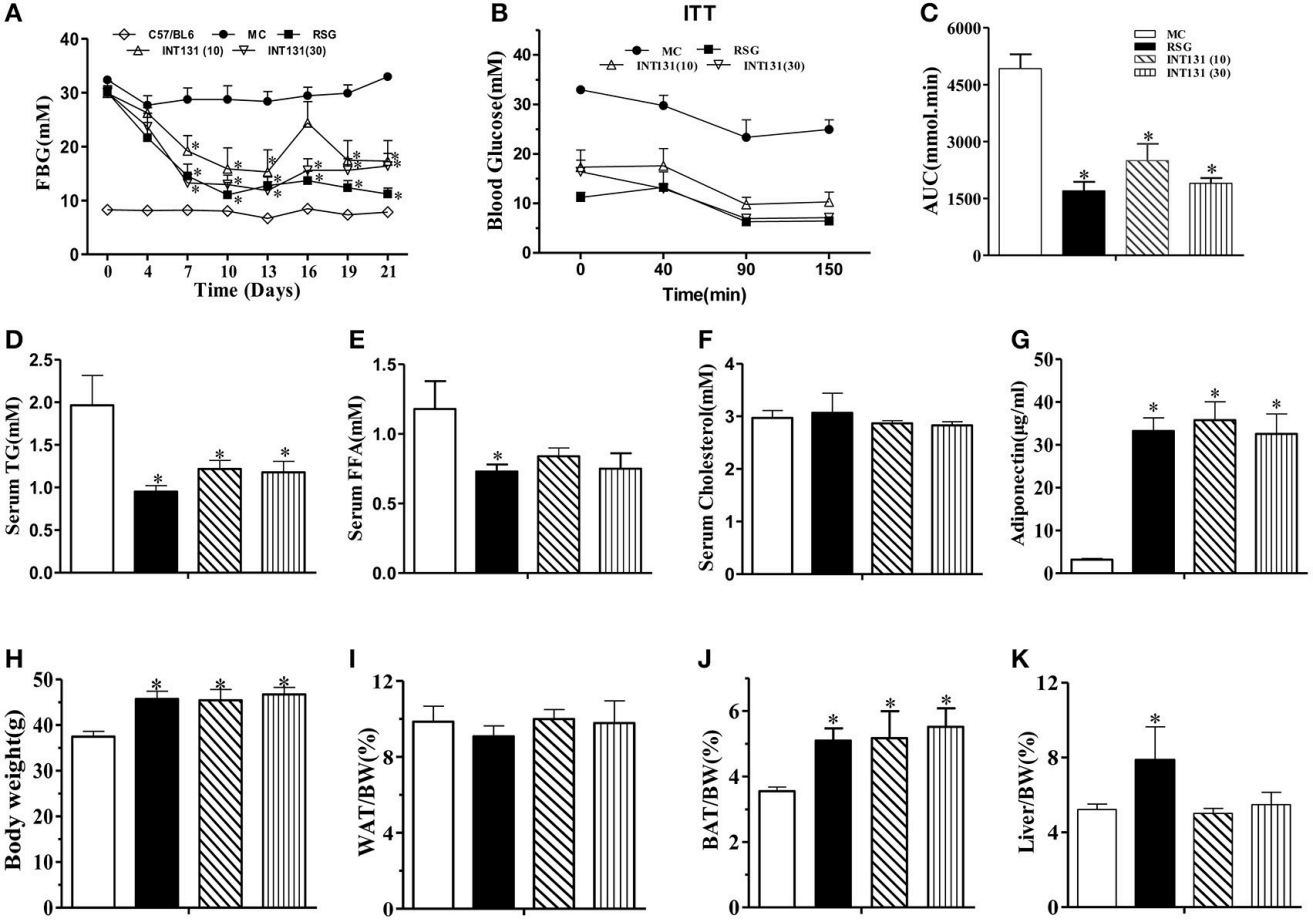
INT131 improves insulin resistance similar to rosiglitazone in db/db mice. **(A)** Fasting blood glucose (FBG) was monitored in male db/db mice every 3 days after treatment with vehicle, 10 mg kg^−1^ rosiglitazone (RSG), 10 mg kg^−1^ INT131 (INT131 10), or 30 mg kg^−1^INT131 (INT131 30). **(B)** The insulin tolerance test (ITT). Insulin (1 U kg^−1^) was administered by i.p. injection to 6-h-fasted db/db mice after 12 days of treatment. **(C)** Area under the curve for the ITT curve. **(D)** Serum TG, **(E)** FFA, **(F)** Cholesterol, and **(G)** Adiponectin. **(H–K)** Body weight, WAT, BAT and liver index determined at the end of the experiment. MC, model control; *n* = 5–6 for each group. Multiple comparisons were performed with one-way ANOVA followed by Tukey's multiple comparison tests. Data are mean ± SE. ^*^p < 0.05 compared with the MC.

We first sought to identify and confirm a dose of INT131 that resulted in equivalent insulin sensitivity to that of 10 mg kg^−^^1^ RSG, a dose commonly used in rodent studies(Chen et al., 2009; Zhang et al., 2013), for further use in our pharmacokinetic assays and molecular studies. As shown in Figure 1A, in diabetic db/db mice, INT131 lowered fasting blood glucose (FBG) in a dose-dependent manner within 1 week of treatment. Overall, 30 mg kg^−^^1^ INT131 exhibited similar potency in terms of its ability to lower FBG, serum TG, and FFA levels and to enhance insulin sensitivity and serum adiponectin content (Figure 1). However, in contrast to the results observed in rats (Motani et al., 2009), 10 and 30 mg kg^−^^1^ of INT131 both produced equal body weight gains compared with that observed after treatment with 10 mg kg^−^^1^ RSG (Figure 1H). However, unlike RSG, which increased the liver and BAT mass indices, INT131 did not affect the hepatic weight index (Figure 1K).

**Figure 2:**
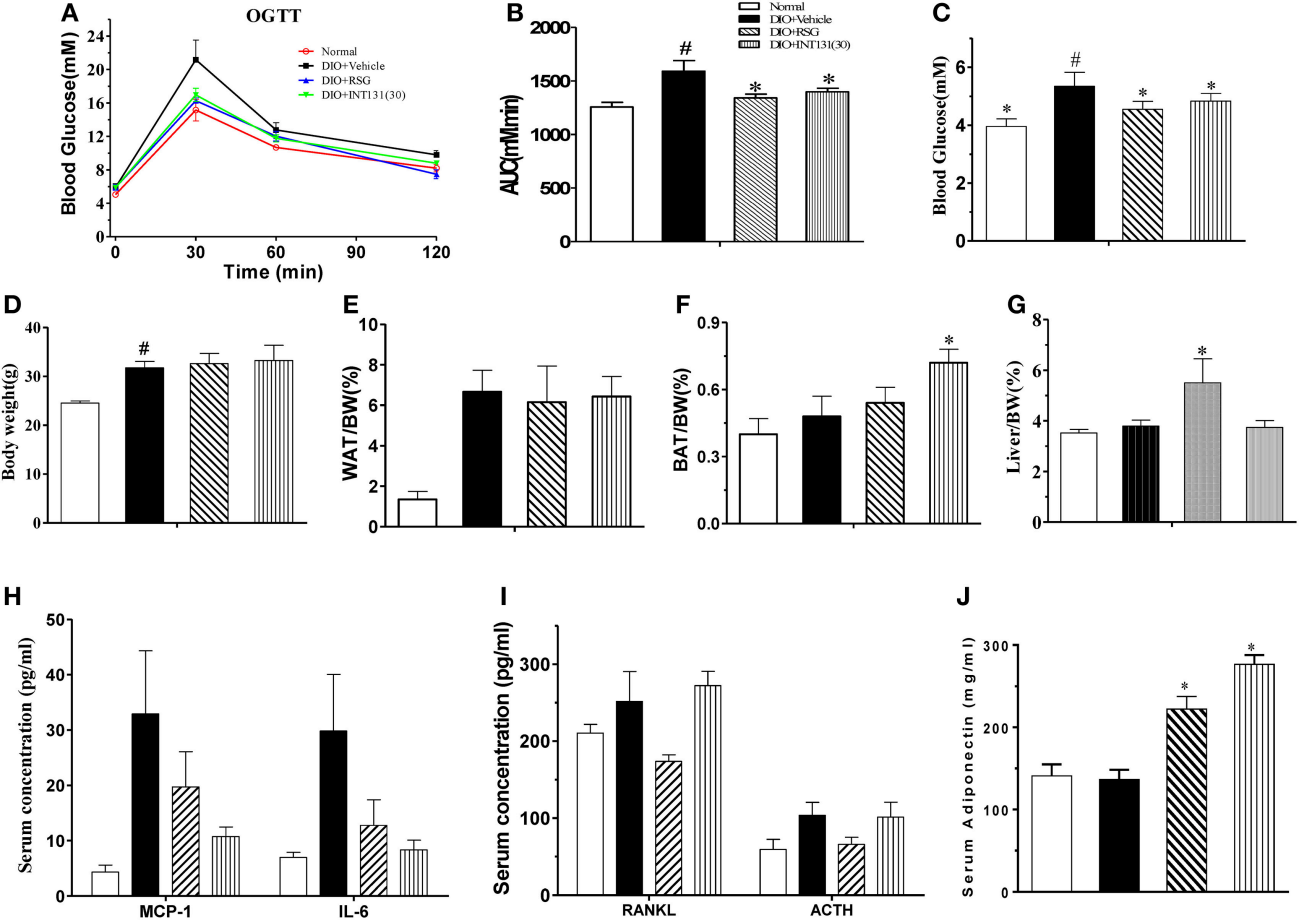
INT131 improves insulin resistance similar to rosiglitazone in DIO mice. **(A)** Oral glucose tolerance test (OGTT) were performed on male DIO mice after treatment with vehicle, 10 mg kg^−1^ rosiglitazone (RSG) or 30 mg kg^−1^ INT131 (INT131 30). Glucose (1 g kg^−1^) was administered by gavage to 12-h-fasted DIO mice. **(B)** Area under the curve for OGTT. **(C)** FBG, **(D–G)** body weight, iWAT, BAT, and liver index. **(H–J)** Serum concentrations of MCP-1, IL-6, RANKL, ACTH, and adiponectin in the DIO mice. MC, model control; *n* = 5–6 for each group. Multiple comparisons were performed with one-way ANOVA followed by Tukey's multiple comparison tests. Data are mean ± SE. ^*^p < 0.05 compared with the vehicle. ^#^*P* < 0.05 compared with normal mice.

Then, the equivalent *in vivo* insulin-sensitizing dose of INT131 was further confirmed in insulin-resistant DIO mice. As observed in the db/db mice, OGTT and FBG detection revealed similar insulin-sensitizing activity after treatment with either 30 mg kg^−^^1^ INT131 or 10 mg kg^−^^1^ RSG (Figures 2A–C). However, INT131 also did not induce an increase in hepatic weight in DIO mice (Figure 2G). Both RSG and INT131 showed no impact on body weight and WAT, whereas they increase the BAT, especially for INT131 (Figures 2D–F). Furthermore, the multiplex detection of serum factors revealed equal elevation of adiponectin and decreased levels of the pro-inflammatory cytokines MCP-1 and IL-6 after treatment with INT131 or RSG (Figures 2H–J). Thus, 30 mg kg^−^^1^ INT131 produces insulin-sensitizing activity equivalent to that of 10 mg kg^−^^1^ RSG but exerts different effects in target tissues.

### INT131 does not induce hepatic steatosis and PPARγ activation in the liver of db/db and DIO mice

**Figure 3:**
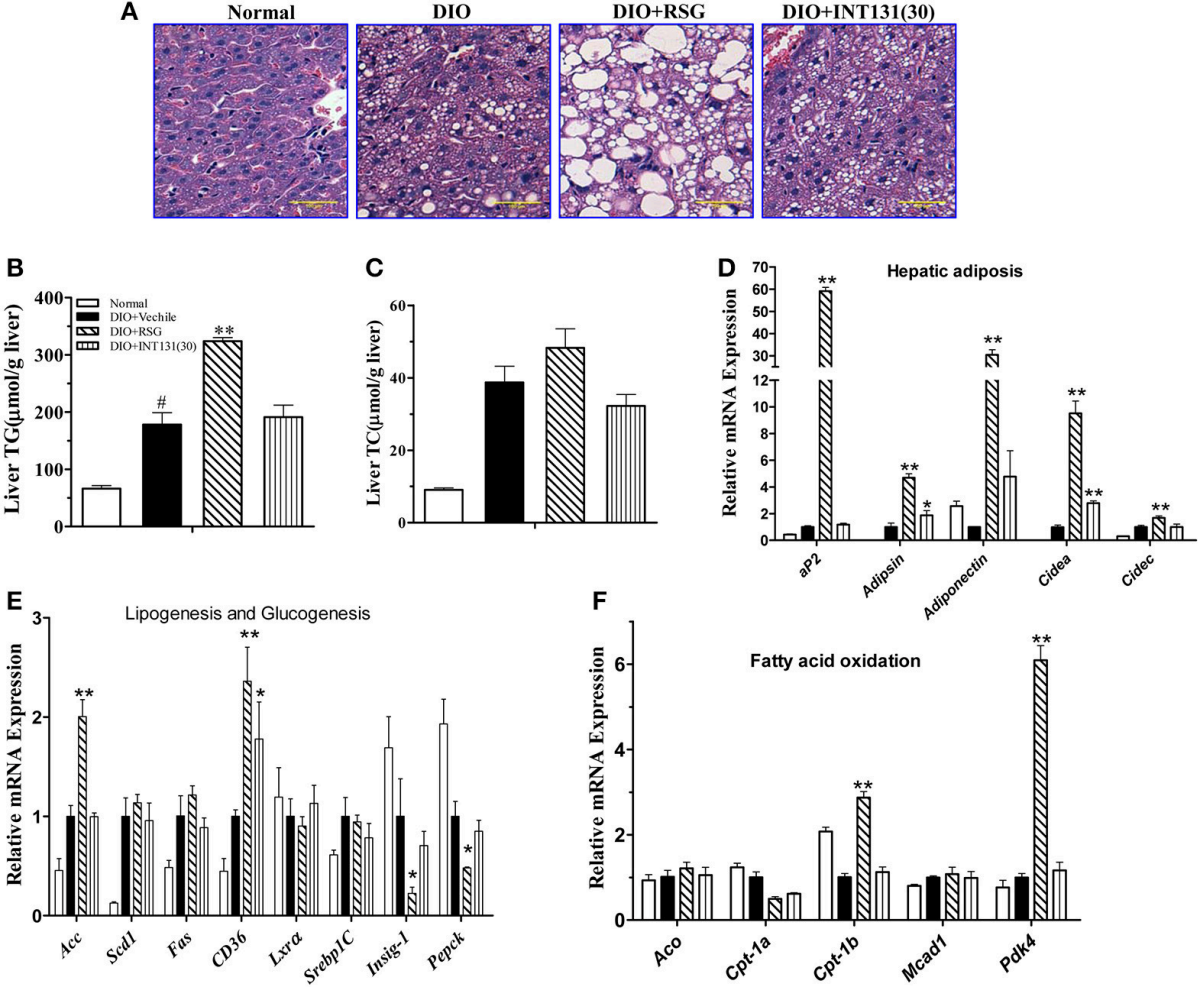
INT131 does not elicit hepatosteatosis or promote PPARγ target gene expression in livers of DIO mice. **(A)** Representative liver HE staining image. **(B,C)** Liver TG and cholesterol concentration (*n* = 5–6 in each group). **(D)** Expression of genes related to hepatic adipogenesis, **(E)** Lipogenesis and glucogenesis, and **(F)** Fatty acid oxidation. *n* = 3 for each group. Multiple comparisons were performed with one-way ANOVA followed by Tukey's multiple comparison tests. Data are mean ± SD. ^*^p < 0.05, ^**^p < 0.01 compared with the vehicle. ^#^*P* < 0.05 compared with normal mice.

RSG-induced hepatomegaly in mice is primarily derived from hepatic steatosis. Hepatic lipid content and morphology analyses have revealed the tissue-specific effects of INT131. As shown in Figure 3A, INT131 had no impact on morphology of hepatocyte and hepatic lipid content, unlike RSG, which dramatically aggravated hepatic hypertrophy and increased lipid accumulation (particularly TG) in hepatocytes, compared with the effects in vehicle-treated DIO mice (Figures 3B,C).

Hepatic PPARγ activation induces the up-regulation of downstream target genes involved in adipogenesis, and lipid metabolism is responsible for RSG-induced hepatomegaly and hepatic steatosis. We next used qPCR to determine whether INT131 affects the expression of these PPARγ target genes in the liver. As expected, RSG robustly up-regulated the expression of the adipocyte-specific genes *aP2, adipsin, adiponectin, cidea*, and *cidec* (Figure 3D), whose ectopic expression in the liver correlated with hepatic adipogenesis and lipid accumulation. In contrast, INT131 did not induce expression of *aP2, adiponectin*, and *cidec*, and induced *adipsin* and *cidea* to a lesser degree than that observed after RSG treatment. RSG dramatically stimulated the expression of genes involved in lipogenesis (*Acc*) and fatty acid uptake (*Cd36*) and inhibited the key gluconeogenesis gene (*Pepck*), whereas INT131 only slightly induced an increase in *Cd36* expression (Figure 3E). Additionally, RSG rather than INT131 specifically induced a marked up-regulation of the expression of fatty acid oxidation genes *Cpt1b* and *Pdk4* (Figure 3F). Similar gene expression patterns were also observed in db/db mice (Supplementary Figure 1). In summary, INT131 does not activate or only weakly activates the expression of PPARγ target genes in the liver.

**Figure 4:**
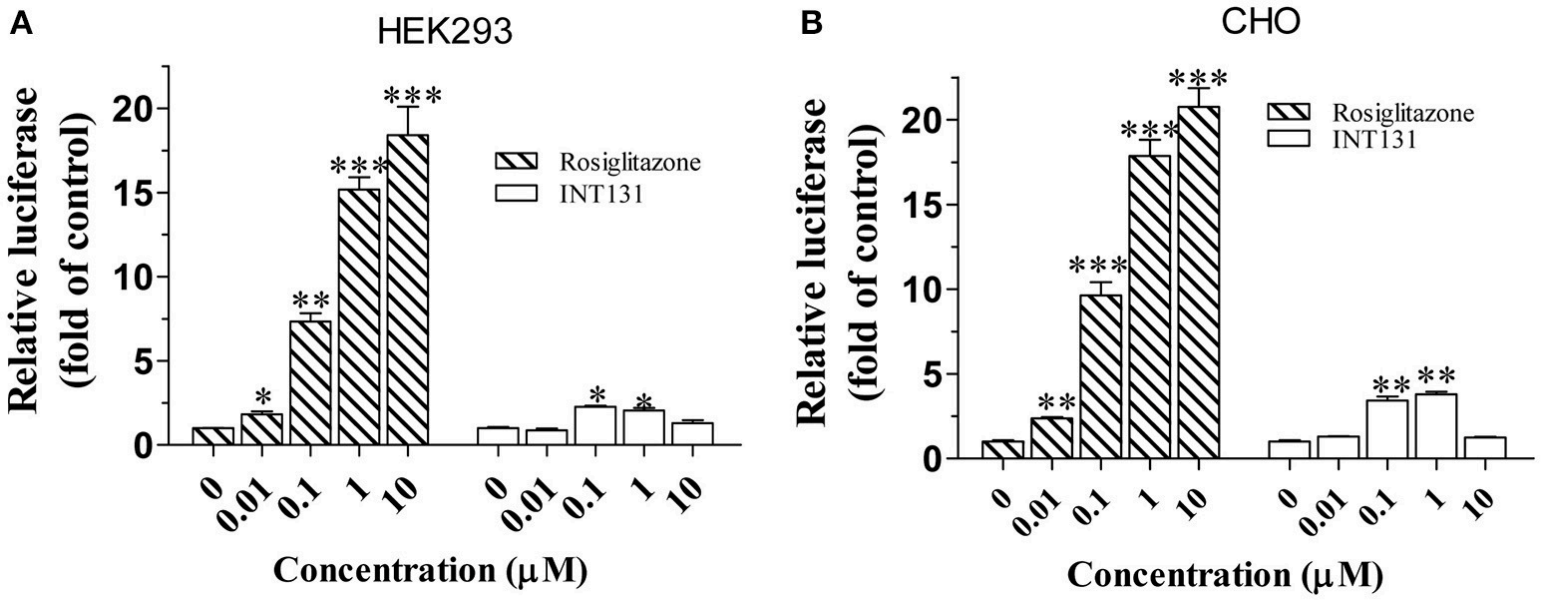
Cofactor recruitment by rosiglitazone and INT131. **(A)** HEK293T and **(B)** CHO cells were transfected with plasmids encoding pACT-hPPARγ2 and pBIND-hMED1 and the reporter construct pG5-luc. After transfection, cells were treated with RSG and INT131 (0.01, 0.1, 1, and 10 μM). The activity of luciferase normalized to that of Renilla, encoded by the plasmid pRL-tk, was used as an internal control. *n* = 3 for each group. Comparisons were determined with unpaired two-tailed Student's *t*-tests. Data are mean ± SD. ^*^p < 0.05, ^**^p < 0.01, ^***^p < 0.001 compared with vehicle control.

As reported, the coactivator mediator subunit MED1 is the only coactivator required for PPARγ-stimulated hepatic steatosis (Bai et al., 2011). Accordingly, a mammalian two-hybrid assay was performed to compare the abilities of INT131 and RSG to recruit MED1 in the HEK293 and CHO cell lines. As shown in Figure 4, RSG recruited substantial amounts of MED1 at a concentration of 0.01 μM (*p* < 0.05 and *p* < 0.01 in HEK293 and CHO cells, respectively), whereas under the same conditions, INT131 induced no MED1 recruitment. Even at the highest concentration (1 μM), INT131 induced much less recruitment than RSG did in both HEK293 and CHO cells (13.5 and 21.2%, respectively, of the effects of RSG at the highest dose). This result indicates the non-hepatic steatosis observed with INT131 is related with weaker hepatic PPARγ activation, which is associated with weak MED1 recruitment.

### INT131 induces adipogenesis, lipogenesis, and PPARγ activation similarly to RSG in the BAT of db/db and DIO mice

**Figure 5:**
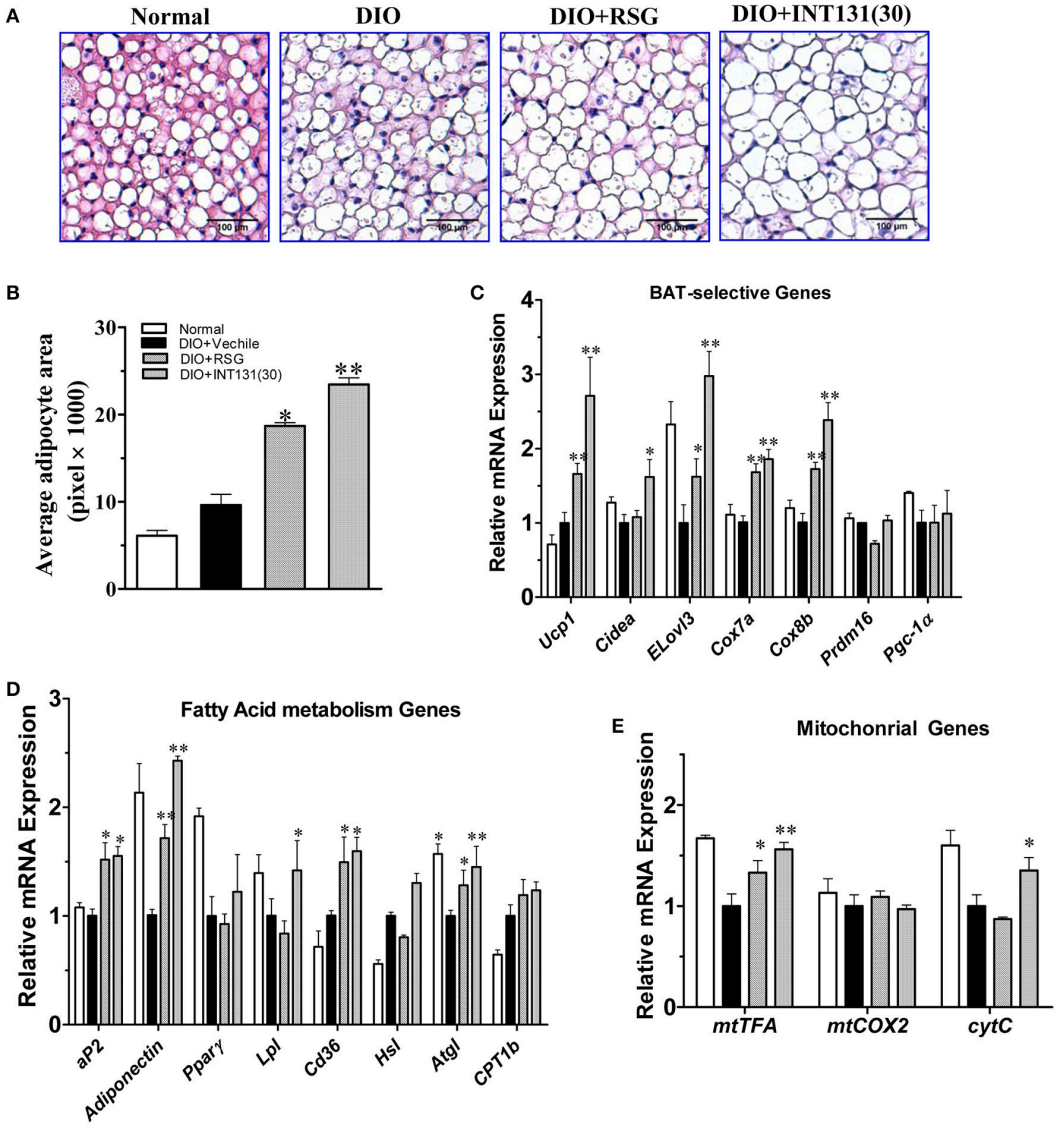
INT131 upregulates BAT-selective gene expression and adipogenesis in BAT of DIO mice. **(A)** Brown adipose tissues of DIO mice treated with vehicle, RSG or INT131 were sectioned and stained with HE, and **(B)** the average adipocyte area was determined. Data are means ± SE. **(C)** Expression of BAT-selective genes and genes related to **(D)** fatty acid metabolism and **(E)** mitochondrial function. *n* = 3 for each group. Multiple comparisons were performed with one-way ANOVA followed by Tukey's multiple comparison tests. Data are mean ± SD. ^*^p < 0.05, ^**^p < 0.01 compared with the vehicle.

PPARγ activation-induced lipid accumulation and brown adipocyte hypertrophy are closely related to BAT-induced weight gain. Consistently with the BAT weight gain observed in DIO mice and similarly to RSG, INT131 induced more severe brown adipocyte hypertrophy, which manifested as increased adipocyte size (Figure 5A) and area (Figure 5B). PPARγ activation in BAT generally leads to the up-regulation of genes involved in thermogenesis, adipogenesis, and lipogenesis. As expected, the results of our qPCR assay indicated that both RSG and INT131 induced substantially up-regulation of BAT marker genes (*Ucp1, Cidea, Elove3, Cox7a*, and *Cox8b*) and of adipogenesis and lipogenesis genes (*aP2, adiponectin, Lpl*, and *Cd36*), although this up-regulation was more pronounced in the INT131 group (Figures 5C,D). Additionally, INT131 and RSG induced similar expression levels of the mitochondrial energy metabolism gene *mtTFA* (Figure 5E). Comparable regulation was also observed in the BAT of db/db mice (Supplementary Figure 2). Thus, on the basis of this gene expression profile, INT131 appears to activate PPARγ in BAT in a manner similar to that of RSG.

### INT131 induces white adipose browning and inhibits inflammation similarly to RSG in both the sWAT and eWAT of db/db and DIO mice

**Figure 6:**
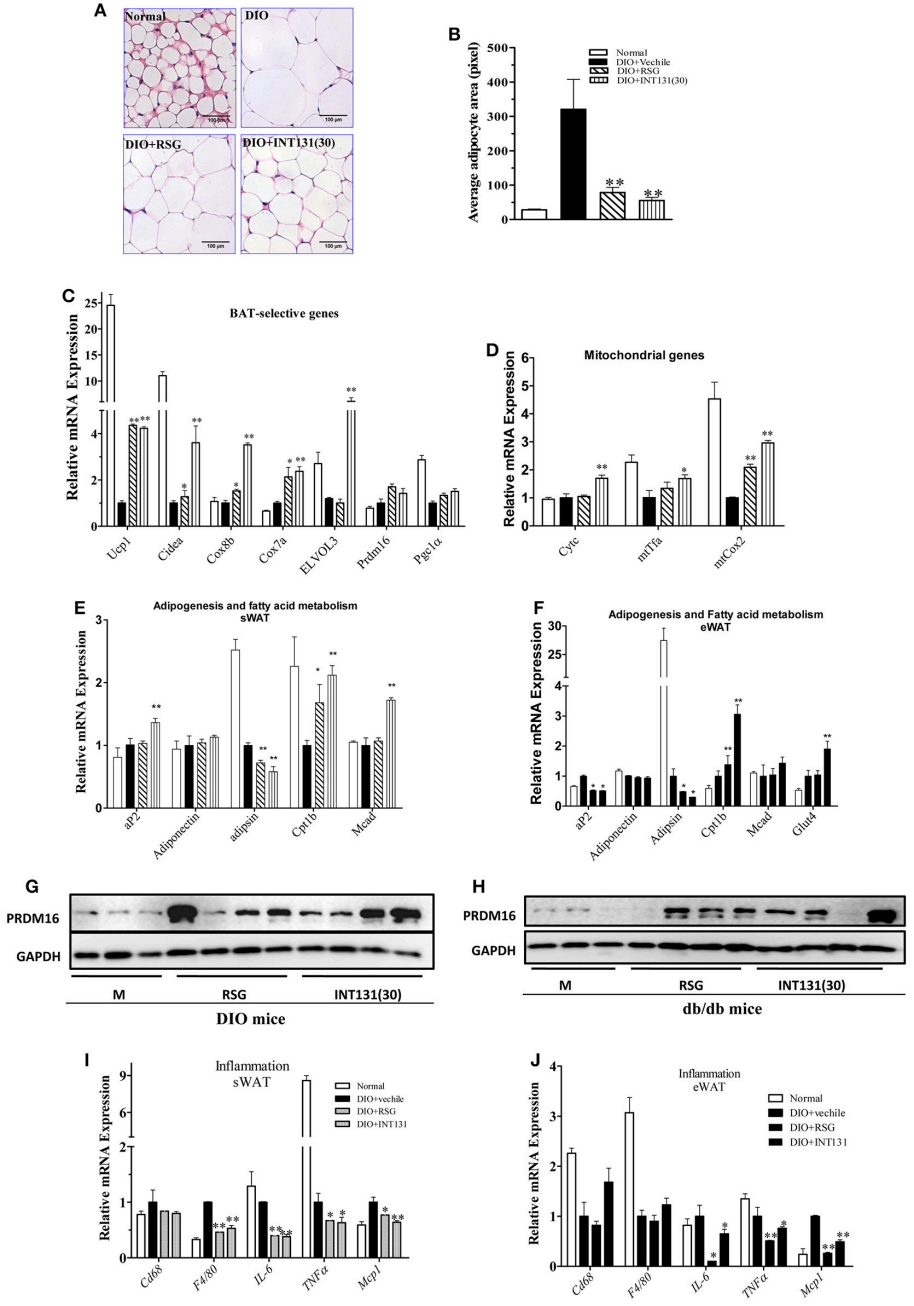
INT131 stimulates WAT browning, up-regulates expression of genes of BAT-selective, adipogenesis, and fatty acid metabolism and inhibits expression of inflammation-related genes in sWAT and eWAT of DIO mice. **(A)** sWATs of DIO mice were sectioned and stained with HE, **(B)** and the average adipocyte area was determined. Data are means ± SE. **(C)** Expression of BAT-selective genes and genes related to **(D)** mitochondrial function, **(E,F)** adipogenesis and fatty acid metabolism. **(G,H)** Detection of PRDM16 protein expression in sWAT. Total lysates of sWAT were obtained from DIO and db/db mice (*n* = 3–4 in each group) and subjected to western blotting. **(I,J)** Expression of inflammation-related genes in sWAT and eWAT. *n* = 3 for each group. Multiple comparisons were performed with one-way ANOVA followed by Tukey's multiple comparison tests. Data are mean ± SD. ^*^p < 0.05, ^**^p < 0.01 compared with the vehicle.

RSG induces WAT trans-differentiation into brite (brown adipocytes in WAT) or beige adipocytes, thus markedly contributing to the insulin-sensitizing activity of this compound; such effects are often lost with many partial agonists. Thus, the white browning effects of INT131 *in vivo* were analyzed. As shown in Figures 6A,B, RSG and INT131 both markedly decreased the sizes of expanded adipocytes in the sWAT of DIO mice, thus suggesting that adipocyte numbers in the WAT increased although the total adipose tissue mass was unchanged. Furthermore, gene expression analysis indicated that INT131 and RSG both up-regulated the expression of BAT-selective genes and mitochondrial genes in the sWAT in DIO (Figures 6C,D) and db/db mice (Supplementary Figure 3), although the effects of INT131 were more pronounced. In addition, INT131 and RSG similarly up-regulated the expression of adipogenesis and fatty acid metabolism genes in both the sWAT and eWAT in DIO and db/db mice (Figures 6E,F).

PRDM16, a key determining factor of BAT, is also a coactivator of PPARγ that activates and regulates BAT-selective gene expression. RSG-induced white browning of WAT depends on the stabilization of the PRDM16 protein. Thus, PRDM16 mRNA and protein levels in the sWAT were determined. Although, neither RSG nor INT131 affected PRDM16 gene expression, both markedly increased PRDM16 protein levels in DIO and db/db mice (Figures 6G,H). Collectively, all morphological and gene and protein expression data suggest the ability of INT131 to induce white browning in the sWAT, similarly to RSG.

The inhibition of macrophage infiltration into the WAT and pro-inflammatory cytokine expression together constitute one of the pharmacological mechanisms underlying RSG-mediated insulin-sensitizing activity. As described above, INT131 and RSG comparably improved *MCP1* and *IL-6* expression in the serum. INT131 and RSG also comparably reduced the levels of macrophage markers *Cd68* and *F4/80*, the pro-inflammatory cytokines *IL-6* and *TNF*α and the chemokine *Mcp1* both in sWAT (Figure 6I) and eWAT (Figure 6J) in DIO and db/db mice (Supplementary Figure 4).

### INT131 induces intramuscular adipocytes similarly to RSG in the skeletal muscles of db/db and DIO mice

**Figure 7:**
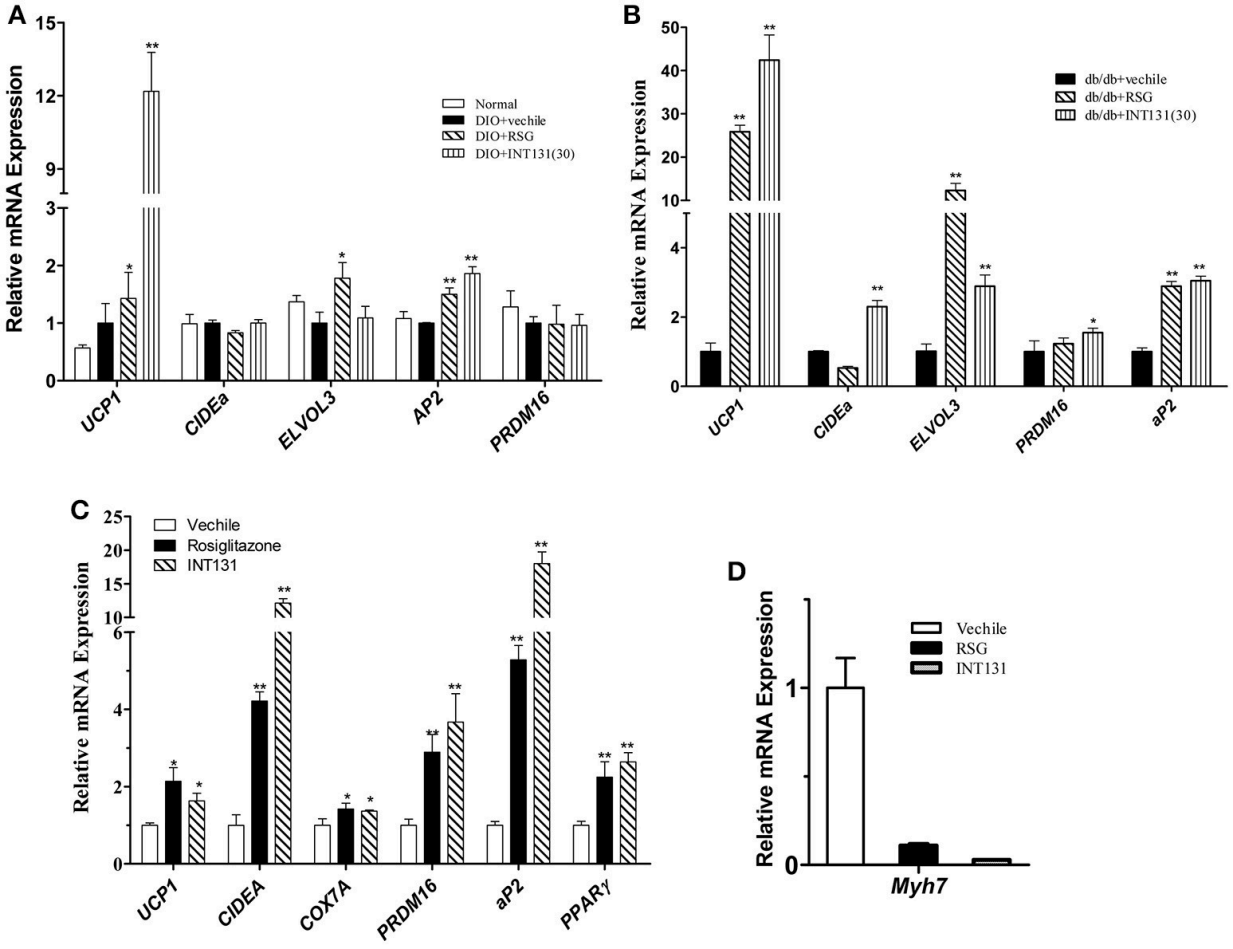
INT131 upregulates BAT-selective and adipogenic gene expression in skeletal muscle of DIO mice. **(A)** Expression of BAT-selective and adipogenic genes in skeletal muscle of DIO and **(B)** db/db mice. **(C)** Expression of BAT-selective and adipogenic genes and **(D)** muscle-specific genes in C2C12 cells; gene expression assay in the C2C12 cells were detected after treatment with 10 μM of RSG or INT131 for 5 days. *n* = 3 for each group. Multiple comparisons were performed with one-way ANOVA followed by Tukey's multiple comparison tests. Data are mean ± SD. ^*^p < 0.05, ^**^p < 0.01 compared with the vehicle.

Muscle is a major organ responsible for insulin-stimulated glucose disposal. RSG enhances glucose uptake, and intramuscular adipocyte and fatty acid metabolism by activating PPARγ. Expression of genes involved in adipogenesis was analyzed in the skeletal muscle to compare INT131-induced PPARγ activation to that of RSG. Notably, INT131 and RSG enhanced *Ucp1, Elove3*, and *aP2* gene expression in both DIO and db/db mice (Figures 7A,B); these genes are specifically expressed in BAT and adipose tissue. According to previous studies, TZDs convert myogenic cells into adipose-like cells by activating PPARγ. To confirm that the ability of INT131 and RSG to stimulate intramuscular adipogenesis effects *in vivo* derived from the direct conversion of myogenic cells, C2C12 cells were continuously exposed to RSG and INT131 at a dosage of 10 μM for 5 d after the cells had reached confluence. Gene expression assay of those responded to adipogenesis and myogenesis indicated that INT131 was able to switch myogenic cells to adipocytes in the same manner as RSG (Figures 7C,D). Thus, INT131 appears to activate PPARγ similarly to RSG in the skeletal muscle.

### INT131 exhibits tissue-specific distribution in insulin target tissues in DIO mice

**Figure 8:**
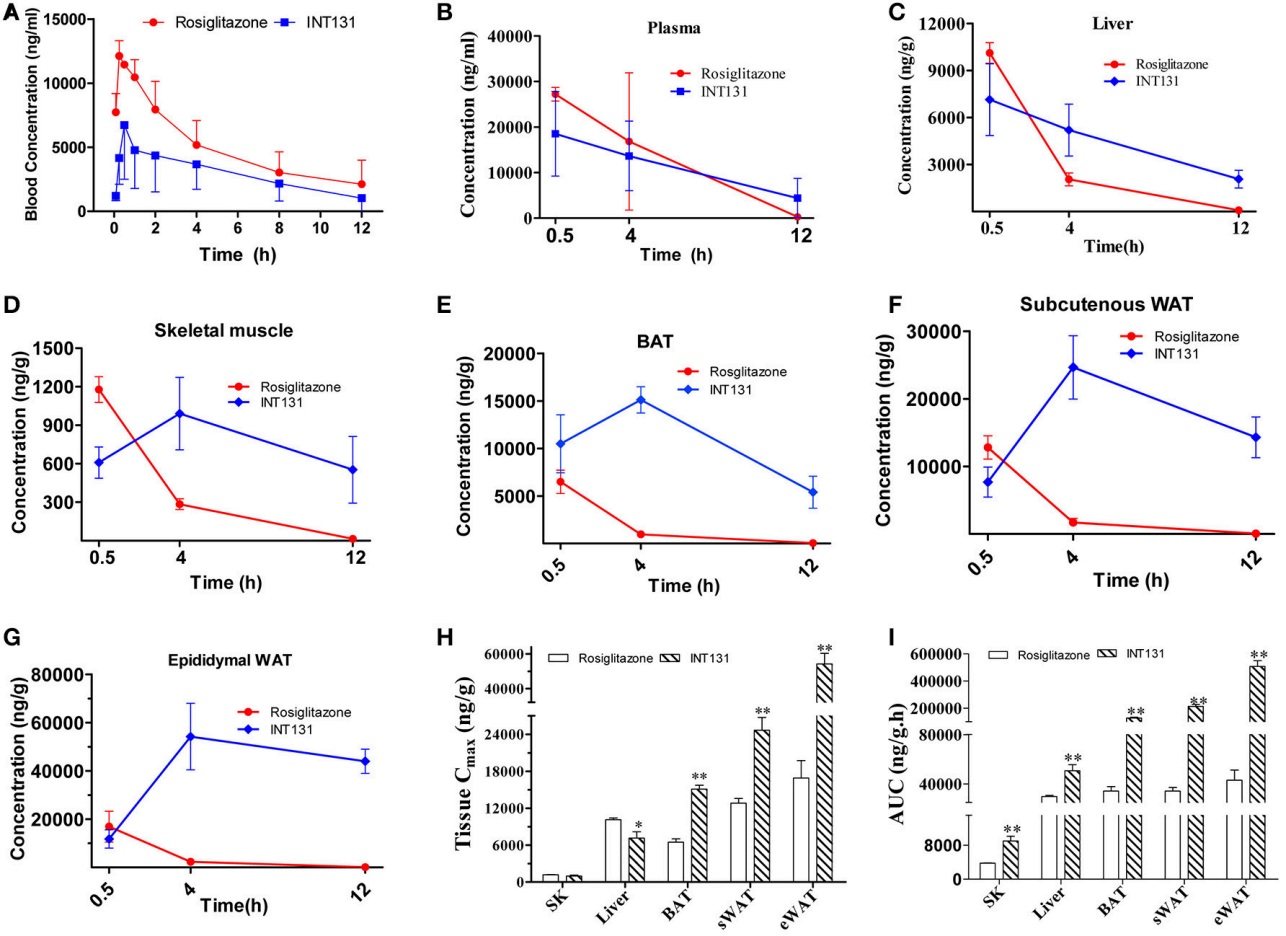
INT131 displays tissue specific distribution in insulin target tissues in DIO mice. **(A)** Blood concentration-time profile of rosiglitazone and INT131 after a single oral gavage with rosiglitazone (10 mg kg^−1^) or INT131 (30 mg kg^−1^). **(B)** Plasma rosiglitazone and INT131 concentration over time and their distribution in **(C)** liver, **(D)** skeletal muscle, **(E)** BAT, **(F)** sWAT, **(G)** and eWAT. **(H,I)** Cmax and AUC value for INT131 and rosiglitazone in these tissues. SK, skeletal muscle. *n* = 4–5 for each group. Comparisons were determined with unpaired two-tailed Student's *t-*tests. Data are mean ± SD. ^*^p < 0.05, ^**^p < 0.01 compared with the vehicle.

The local concentration of a drug (including the Cmax and residency time) at the site of action determines a drug's potency and also dramatically affects its side effects. To further analyse whether the differentiated and selective PPARγ activation initiated by INT131 in different insulin target tissues was related to their differential tissue contents, INT131 and RSG distribution in the primary insulin target tissues of DIO mice were assayed in detail. Plasma and tissue samples, including WAT, BAT, liver, and skeletal muscle, were collected from DIO mice at 0.5, 4, and 12 h after oral gavage with RSG or INT131 according to the blood concentration-time profiles generated in our pre-test, in which the average residency times were 4.03 ± 0.83 and 4.4 ± 1.17 h for RSG and INT131, respectively (Figure 8A).

The differential distribution patterns of INT131 and RSG in all analyzed insulin target tissues are shown in Figures 8B–I. Although, the plasma concentrations of both compounds decreased over time in a similar manner after oral administration, the concentration of RSG in all assayed tissues also declined over time after reaching a peak (Cmax) at 0.5 h; by 12 h, the concentration in these tissues was almost negligible. In contrast, the concentrations of INT131 in skeletal muscle and adipose tissues (eWAT, sWAT, and BAT) increased first and peaked at 4 h, then decreased very slowly over time (Figures 8B–G), whereas the plasma and hepatic concentration of INT131 continually decreased after it reached a peak at 0.5 h. Both the content and elimination of INT131 and RSG in the liver were similar. Additionally, when we compared the Cmax values for RSG and INT131 in the same tissues, we found that they both had similar Cmax values in skeletal muscle (Figure 8G), whereas the Cmax for INT131 was lower (0.71-fold of RSG) in the liver, and the Cmax values for INT131 in all assayed adipose tissues (BAT, sWAT, and eWAT) were ~2–3 times greater than those of RSG (Figure 8H). The different times required by each compound to reach Cmax may be related to their differential absorption efficacies or differential protein binding and dissociation rates.

Moreover, the elimination of INT131 from all assayed tissues was much slower than that of RSG, particularly in adipose tissues and skeletal muscle. At 12 h after dosing, the concentration of INT131 was ~440, 209, 180, and 40 times that of RSG in eWAT, sWAT, BAT, and skeletal muscle, respectively (Figures 8D–G). Calculation of the AUC_0−12h_ on the basis of the concentration-time curve indicated that mice treated with INT131 consistently demonstrated higher AUC_0−12*h*_ values (representing the total exposure amount) in all analyzed insulin target tissues, specifically 11.82-, 6.20-, 3.71-, and 2.37-fold higher than those of RSG in eWAT, sWAT, BAT, and skeletal muscle, respectively (Figure 8I). Thus, the residency times of INT131 in all adipose tissues and skeletal muscle were much longer than those of RSG. Taken together, INT131 was observed to accumulate in wanted, PPARγ-expressing target tissues like adipose tissue (and skeletal muscle) much better than RSG, and to a lower degree in the liver. Therefore, the unique characteristics in tissue distribution of INT131 underlined its tissue-specific PPARγ activation.

## Discussion

In current study, the relationship between the tissue-selective effects and differential tissue distribution of the sPPARγM INT131 and the classical PPARγ full agonist RSG was brought forward from a brand new view and elucidated comparatively under equivalent dose of the two molecules in both insulin-resistant and diabetic mice with pharmacokinetic assay. The tissue-specific modulation of representative PPARγ target genes by INT131 relative to RSG was observed in comparison. In contrast to RSG, INT131 did not aggravate hepatic steatosis or affect hepatic adipogenic gene expression, although it did generate equivalent or more potent PPARγ activation and regulation of downstream target genes in adipose tissues and skeletal muscle. Most importantly, the differential tissue-specific PPARγ regulation of INT131 and RSG was demonstrated for the first time to be closely related to the higher distribution of INT131 in major insulin target tissues, including adipose tissues and skeletal muscle, although less recruiting of coactivator MED1 might also be involved in this process.

We first confirmed a dose of INT131 that exerted an insulin-sensitizing effect equivalent to that of 10 mg kg^−1^ RSG. According to our data and other reports, the maximum potency of INT131 in the cell-based PPARγ transactivation assay in HEK293 cells was approximately one-third of RSG's (Supplementary Figure 4). Functionally, we and others also confirmed the weaker effects of INT131 in promoting the differentiation of mouse 3T3-L1 preadipocytes (Motani et al., 2009), and the equivalent dose of INT131 was ~3- to 5-fold of RSG's. Thus, on basis of the *in vitro* results and the prior studies in rodents, in which 3 mg kg^−1^ day^−1^ INT131 was observed to initiate blood glucose lowering effects identical to those obtained with 1 mg kg^−1^ RSG in diabetic Zucker (fa/fa) fatty rats, two doses (10 and 30 mg kg^−1^ day^−1^) of INT131 were examined in diabetic db/db mice first and then corroborated in the insulin-resistant DIO mice. Consistent with the *in vitro* results, 30 mg kg^−1^ INT131 generated equivalent insulin-sensitizing efficacy as 10 mg kg^−1^ RSG, the dose of INT131 is about 3-fold lower than that of RSG, same as above mentioned in the rats. Moreover, in DIO mice, the gene expression assay confirmed equivalent or more potent PPARγ activation and regulation of downstream target genes expression by INT131 in adipose tissues (including sWAT and BAT) and skeletal muscle. Thus, 30 mg kg^−1^ INT131 exerts pharmacodynamic effects equivalent to those obtained with 10 mg kg^−1^ RSG by selectively and specifically regulating PPARγ target gene expression in adipose tissues and skeletal muscle.

Studies investigating general and tissue-specific PPARγ deletion in mice have revealed the contribution from PPARγ activation in all insulin target tissues to the insulin-sensitizing effects (Gavrilova et al., 2003; He et al., 2003; Hevener et al., 2003). However, only PPARγ in adipose tissue is indispensable to maintain whole-body insulin-sensitizing activity (Sugii et al., 2009). PPARγ activation in liver contributes to hepatic steatosis and is not indispensable for insulin sensitization, depending on the diverse rodent backgrounds studied (Gavrilova et al., 2003). Additionally, hepatic PPARγ expression is elevated in fatty liver (Memon et al., 2000). According to our data, in contrast to RSG, INT131 did not exacerbate hepatic hypertrophy and hepatic steatosis and exerted reduced effects on the hepatic expression of adipogenic and lipogenic genes, consistently with the phenotype observed in genetic PPARγ liver-null mice and further indicating INT131 did not stimulate PPARγ activation in the liver. This finding may be related to the specific pharmacokinetics of INT131, particularly its differential tissue distribution. INT131 had a lower Cmax (7047.7 ng g^−1^) in liver than that of RSG (10121.4 ng g^−1^), although its total exposure dose (AUC) was slightly higher (1.7-fold that of RSG; Figures 8H–I). However, the calibrated AUC of INT131 in liver was <1.2-fold that of RSG because of its higher molecular weight (357.4 vs. 514.2). Thus, this dose of INT131 appears to be insufficient to activate hepatic PPARγ because its potency is only ~1/3 that of RSG (Supplementary Figure 4). Meanwhile, it can be seen that the Cmax of INT131 in the liver was only ~1/2–1/7 of those in adipose tissues. Furthermore, the residency time of INT131 in liver was also substantially shorter, and the concentration in liver dropped to almost 1/4, 1/8, and 1/16 of that in the BAT, sWAT, and eWAT, respectively, at 12 h after administration (Figure 8C). Additionally, the recruitment of different co-activators may also be involved in the regulation of INT131-mediated tissue-specific PPARγ activation. INT131 recruited lower levels of MED1 in our cell-based reporter assay, consistently with previously reported data obtained from an *in vitro* molecular FRET assay (Motani et al., 2009). The coactivator mediator subunit MED1 is the only coactivator that is required for PPARγ-stimulated adipogenic hepatic steatosis in mice (Bai et al., 2011). Moreover, in our study, INT131 produced an insulin-sensitizing activity similar to that of RSG in both db/db and DIO mice, although INT131 initiated lower PPARγ activation in the liver. Thus, hepatic PPARγ activation is not indispensable for whole-body insulin-sensitizing action but contributes to the side effects of fatty liver in mice.

Adipose tissue had been recognized as a key tissue in maintaining systemic metabolic and energy homeostasis, where PPARγ is expressed at its highest level and acts as a master regulator of adipocyte biology. Targeted deletion of PPARγ in adipose tissue had been found to result in lipodystrophy or no visible adipose tissue, dyslipidaemia and adipokine deregulation, and extreme insulin resistance (He et al., 2003; Jones et al., 2005; Wang et al., 2013). In a gain-of-function mouse model, specific adipose PPARγ activation is sufficient to enhance whole-body insulin sensitivity and improves adipokine levels, inflammation, and lipid profiles (Sugii et al., 2009). In current study, similarly to RSG, INT131 stimulated PPARγ activation and regulated the expression of corresponding downstream target genes, including those involved in adipogenesis, lipogenesis, thermogenesis, and inflammation, and improved adipokines level in both the WAT and BAT. Surprisingly, in contrast to previous report that full PPARγ agonism is required to induce a brown fat gene programme (Ohno et al., 2012), the sPPARγM INT131 also induced a parallel brown fat gene programme similar to that initiated by RSG in the DIO mice. Moreover, the PRDM16 protein, a critical regulator of brown fat development and function, was stabilized by INT131 in a manner similar to that of RSG (Seale et al., 2008). In summary, our study provides the first report that the sPPARγM INT131 induces white-to-brown fat transition as efficiently as the full PPARγ agonist RSG under condition of equivalent dose, suggesting full PPARγ agonism is not required for PPARγ ligands to induce white browning and also signifying that pure adipose PPARγ activation is sufficient to achieve insulin-sensitizing activity.

The higher Cmax and the much longer residency time (AUC_0−12h_), as well the relatively lower elimination speed of INT131 in all adipose tissues and skeletal muscle revealed the predominant distribution of INT131 in adipose tissues and skeletal muscle compared with that of RSG. This distribution characteristic of INT131 coincides with the distribution manner of its target PPARγ (most abundant in adipose tissues), thereby potentially ensuring sustainable PPARγ activation of INT131 specifically in these tissues. Which strongly suggested a tight correlation exists between the potent INT131-mediated tissue selective PPARγ activation and the tissue-specific distribution of INT131. Structural and physiochemical analysis indicate the relatively higher logP (4.38) of INT131 than that of RSG (3.21) potentially determined the higher lipophilicity of INT131, and thus the selective tissue distribution. Additionally, the different PPARγ activation manner also can't be excluded, as the differential structure of INT131 with RSG determines its non-identical binding mode with the ligand-binding pocket of PPARγ receptor, although it partly overlapped with that of TZD's, but INT131 does not directly contact with the binding site associated with full PPARγ agonism (Kintscher and Goebel, 2009; Motani et al., 2009).

Overall, tissue-selective PPARγ activation is an important and specific means of achieving sPPARγM-mediated insulin sensitization with minor side effects, and most importantly, tissue-selective distribution underlies the tissue-selective PPARγ activation of sPPARγM by INT131.

## Author contributions

Conceived and designed the experiments: LW and HL. Performed the experiments: XX, WC, NZ, MY, CX. Analyzed the data: XX, WC, LW, and HL. Contributed reagents: ZZ. Wrote the paper: XX, WC, and LW. All authors have given approval to the final version of the manuscript.

### Conflict of interest statement

The authors declare that the research was conducted in the absence of any commercial or financial relationships that could be construed as a potential conflict of interest.

## Supplementary material

Supplementary Figure 1 INT131 does not elicit PPAR? target genes expression in liver of db/db mice. **(A)** Expression of genes responding to hepatic adipogenesis, **(B)** lipogenesis and glucogenesis, **(C)** fatty acid oxidation. n = 3 for each group. Multiple comparisons were performed with one-way ANOVA followed by Tukey's multiple comparison tests. Data are mean ± SD. ^*^p < 0.05, ^**^p < 0.01 compared with vehicle.

Supplementary Figure 2 INT131 upregulates BAT-selective, adipogenesis genes expression in BAT of db/db mice. **(A)** Expression of genes related to BAT-selective, **(B)** fatty acid metabolism, and **(C)** mitochondrial function. *n* = 3 for each group. Multiple comparisons were performed with one-way ANOVA followed by Tukey's multiple comparison tests. Data are mean ± SD. ^*^*p* < 0.05, ^**^*p* < 0.01 compared with vehicle.

Supplementary Figure 3 INT131 stimulates WAT browning, up-regulates BAT-selective, adipogenesis and fatty acid metabolism and inhibits inflammation genes expression in sWAT and eWAT of db/db mice. **(A)** Expression of genes responding to BAT-selective, **(B)** mitochondrial function, **(C,D)** adipogenesis and fatty acid metabolism. **(E,F)** expression of genes responding to inflammation in sWAT and eWAT. *n* = 3 for each group. Data are mean ± SD. Multiple comparisons were performed with one-way ANOVA followed by Tukey's multiple comparison test. ^*^*p* < 0.05, ^**^*p* < 0.01 compared with vehicle.

Supplementary Figure 4 PPARγ transactivation by INT131 and RSG. HEK293T cells were cotransfected with pCMX-Gal4-PPARγ-LBD and tk- UAS × 4Luc. After transfection, cells were treated with RSG or INT131 at various concentrations. Luciferase activity normalized to renilla activity encoded by the plasmid pRL-tk was used as an internal control. Data are mean ± SE (*n* = 3).

Supplementary Figure 5 Structure of compound **(A)** INT131 (2, 4-dichloro-N-[3, 5-dichloro-4-(quinolin- 3-yloxy) phenyl] benzene sulfonamide) and **(B)** Rosiglitazone(5-[[4-[2-[methyl(pyridin-2-yl)amino]ethoxy]phenyl]methyl]-1,3-thiazolidine-2,4-dione).

Supplementary Table 1 Primer sequences used in qPCR.
