# Supplementary material for: Selective Tissue Distribution Mediates Tissue-Dependent PPARγ Activation and Insulin Sensitization by INT131, a Selective PPARγ Modulator
Source: Front Pharmacol. 2017 May 30;8:317. doi: 10.3389/fphar.2017.00317 (PMC5447729; doi:10.3389/fphar.2017.00317)
Supplement: Supplementary Figure 1 — INT131 does not elicit PPAR? target genes expression in liver of db/db mice. (A) Expression of genes responding to hepatic adipogenesis, (B) lipogenesis and glucogenesis, (C) fatty acid oxidation. n = 3 for each group. Multiple comparisons were performed with one-way ANOVA followed by Tukey's multiple comparison tests. Data are mean ± SD. *p < 0.05, **p < 0.01 compared with vehicle. [file DataSheet1.pdf]

Supplementary data

Supplementary fig. 1

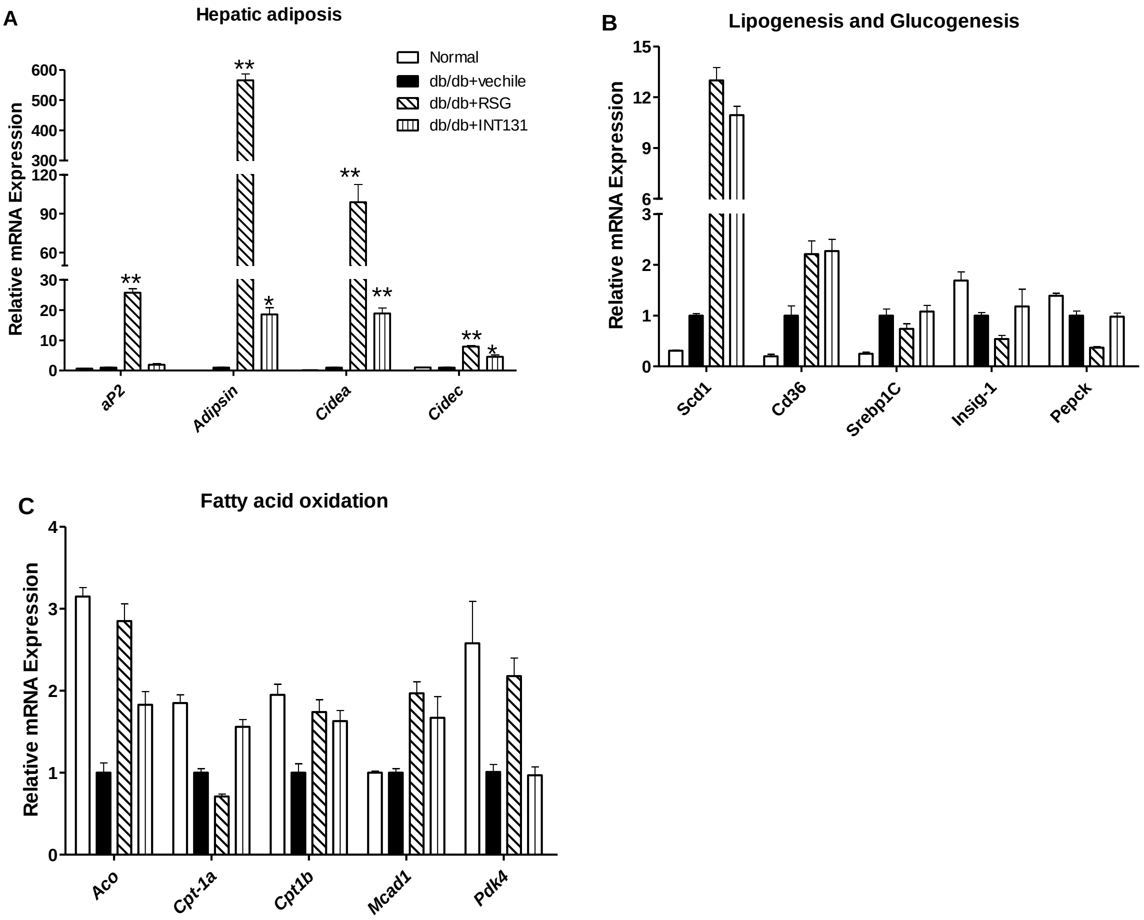

Supplementary fig. 2

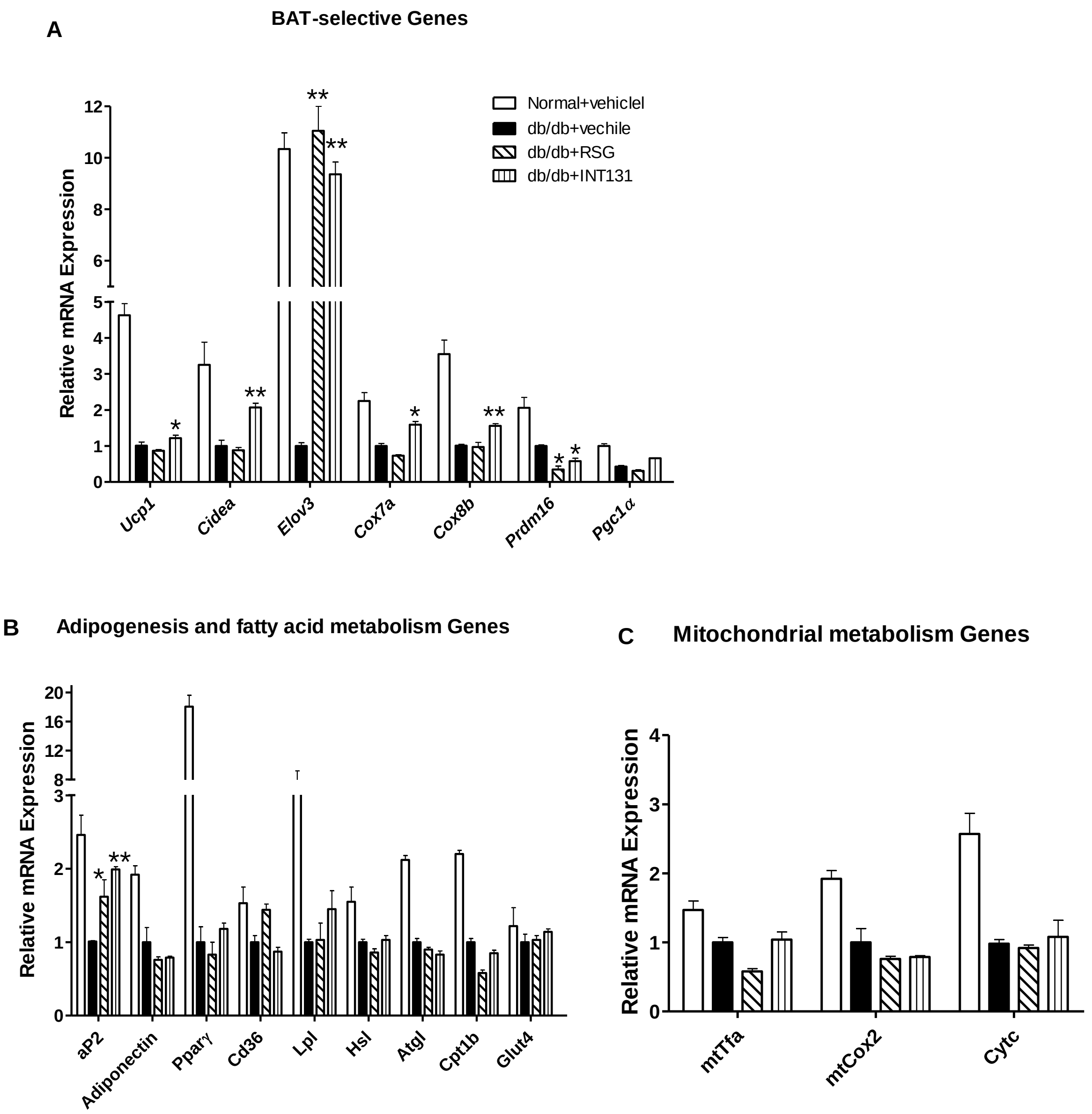

Supplementary fig. 3

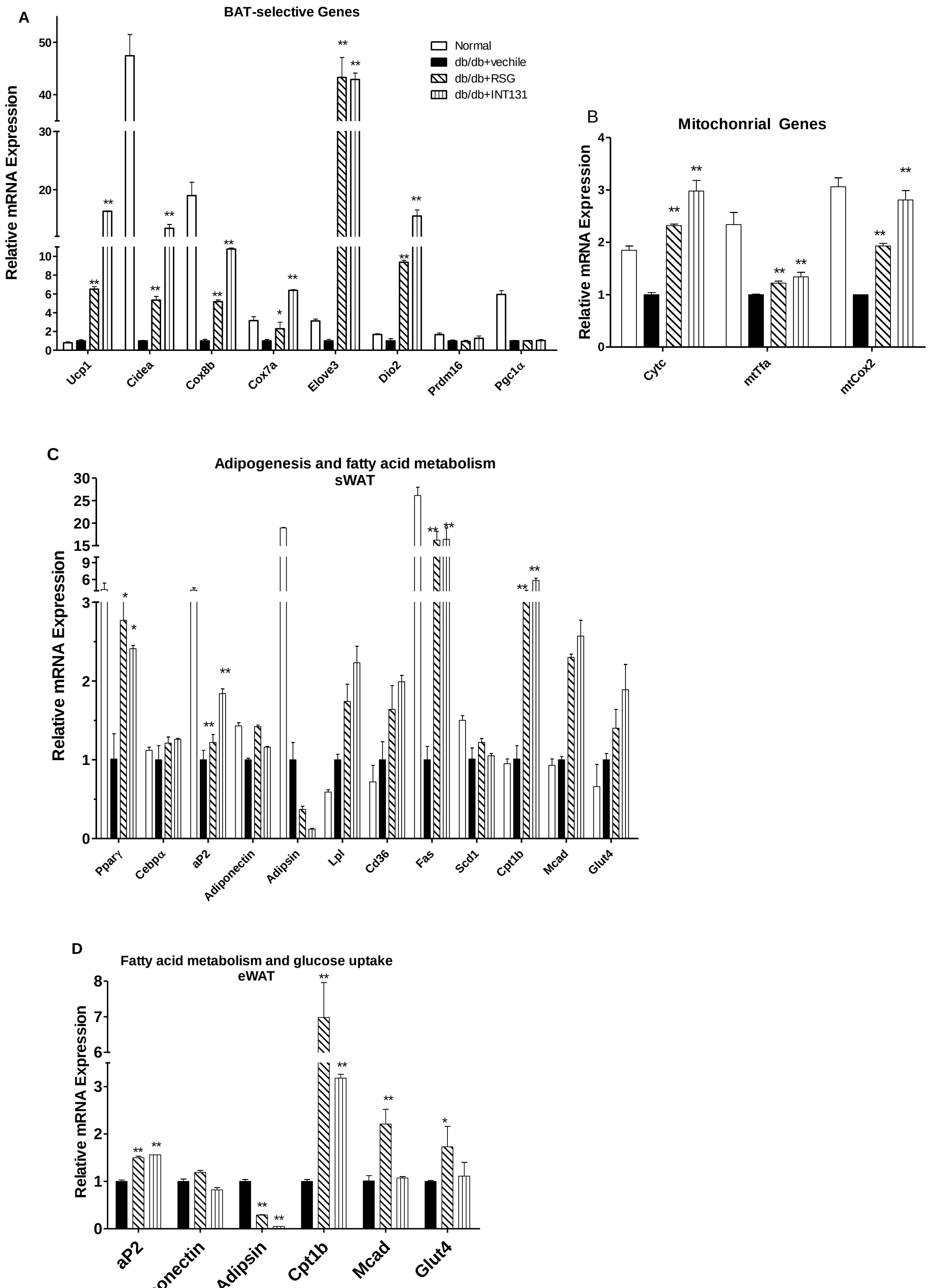

E

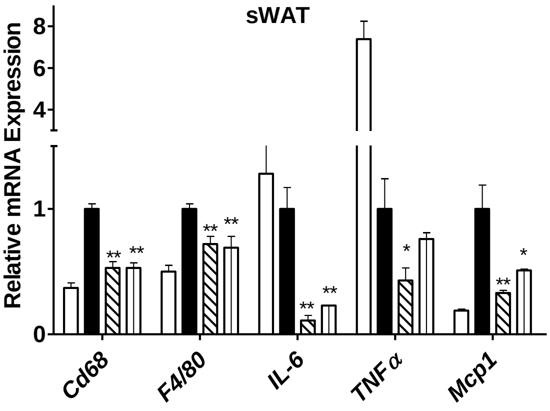
Inflammation
sWAT
Relative mRNA Expression
8
6
4
1
0
Cd68
F4/80
IL-6
TNFα
Mcp1

F

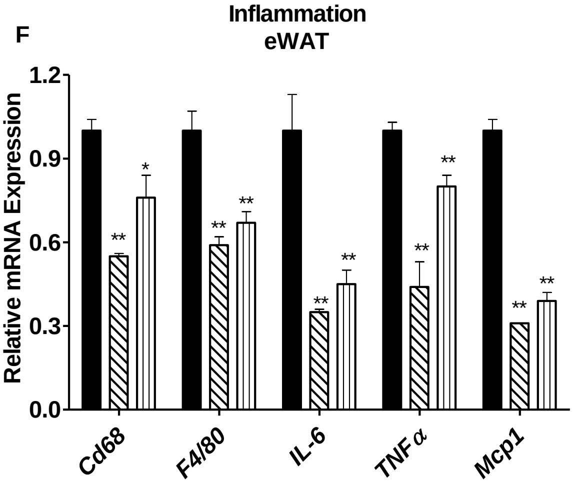
Inflammation
eWAT
Relative mRNA Expression
1.2
0.9
0.6
0.3
0.0
Cd68
F4/80
IL-6
TNFα
Mcp1

Supplementary fig. 4

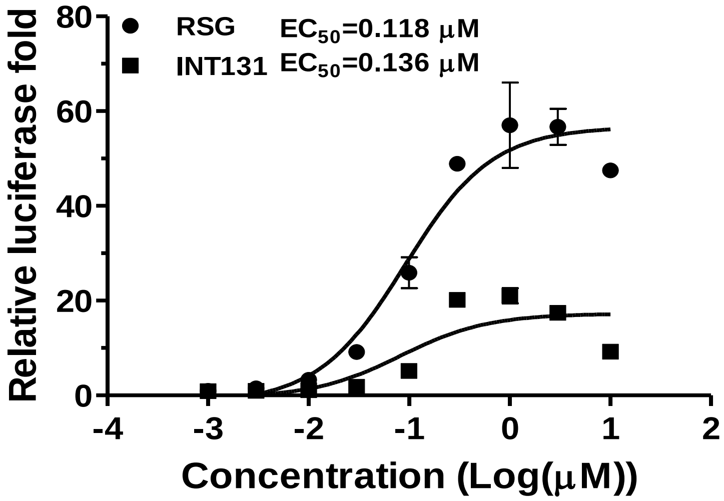

Supplementary fig. 5

A

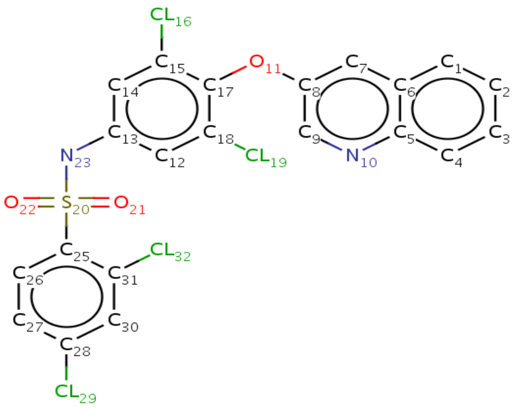

B

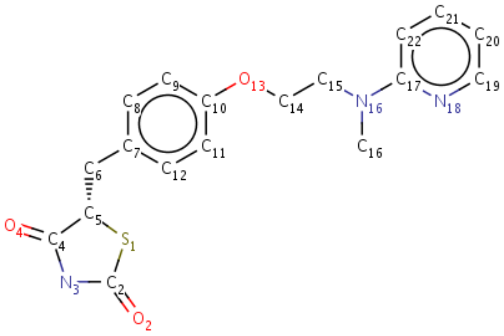

Supplementary table 1 Primer sequences used in qPCR

Gene	Forward primer	Reverse primer
aP2	AAGGTGAAGAGCATCATAACCCT	TCACGCCTTTCATAACACATTCC
adiponectin	GGAGAGAAAGGAGATGCAGGT	CTTTCCTGCCAGGGGTTC
adipsin	CATGCTCGGCCCTACATGG	CACAGAGTCGTCATCCGTCAC
Cidec	ATGGACTACGCCATGAAGTCT	CGGTGCTAACACGACAGGG
Acc	CCCGCTTCTTCAACTTGCT	ATTGGGCACCCCAGAGCTA
Scd1	TTCCCTCCTGCAAGCTCTAC	CAGAGCGCTGGTCATGTAGT
Fas	GCTGGCATTCGTGATGGAGTCGT	AGGCCACCAGTGATGATGTAACTCT
Cd36	AAGCTATTGCGACATGATT	GATCCGAACACAGCGTAGAT
LXR α	GAAAGAGCCTCCAGGGTGA	CCCTGGACATTACCAAGACAC
Insig-1	CACGACCACGTCTGGAACTAT	TGAGAAGAGCACTAGGCTCCG
Srebp-1c	GGTTTTGAACGACATCGAAGA	CGGGAAGTCACTGTCTTGGT
Pepck	ATGTGTGGGCGATGACATT	AACCCGTTTTCTGGGTTGAT
Aco	GCCCAACTGTGACTTCCATC	GCCAGGACTATCGCATGATT
Cpt-1a	GACTCCGCTCGCTCATTC	TCTGCCATCTTGAGTGGTGA
Cpt-1b	TGCCTTTACATCGTCTCCAA	GGCTCCAGGGTTCAGAAAGT
Mcad	TGTCGAACACAACACTCGAAA	CTGCTGTTCCGTCAACTCAA
Pdk4	CGCTTAGTGAACACTCCTTCG	CTTCTGGGCTCTTCTCATGG
Cd68	CTTCCCACAGGCAGCACAG	AATGATGAGAGGCAGCAAGAGG
F4/80	CTTGGCTATGGGCTTCCAGTC	GCAAGGAGGACAGAGTTTATCGTG
TNF α	CCAGACCCTCACACTCAGATC	CACTTGGTGGTTTGCTACGAC
Mcp1	CATCCACGTGTTGGCTCA	GATCATCTTGCTGGTGAATGAGT
IL-6	CCAGAGATACAAAGAAATGATGG	ACTCCAGAAGACCAGAGGAAAT
Ucp1	GGCCTCTACGACTCAGTCCA	TAAGCCGGCTGAGATCTTGT
Cidea	TGCTCTTCTGTATCGCCCAGT	GCCGTGTTAAGGAATCTGCTG
Cox7a	CTGAGGACGCAAAATGAGG	TGGCTTCTGGTAGATGAGCTAAA
Cox8b	GAACCATGAAGCCAACGACT	GCGAAGTTCACAGTGGTTCC
Elovl3	TTCTCACGCGGGTTAAAAATGG	GAGCAACAGATAGACGACCAC
Prdm16	CCCCACATTCCGCTGTGAT	CTCGCAATCCTTGCACTCA
Pgc-1α	AAAGGGCCAAGCAGAGAGA	GTAAATCACACGGCGCTCTT
Hsl	GCGCTGGAGGAGTGTTTTT	CCGCTCTCCAGTTGAACC
Atgl	GAGGGCCAAGAGGAAATTG	CAGTTCCACCTGCTCAGACA
Lpl	CTGGTGGGAAATGATGTGG	TGGACGTTGTCTAGGGGGTA
mtTFA	CGATTTTCCACAGAACAGCT	CCATCAGCTGACTTGGAGTT
MtCOX2	AATTGCTCTCCCCTCTCTACG	GTAGCTTCAGTATCATTGGTGC
CytC	CCAAATCTCCACGGTCTGTTC	ATCAGGGTATCCTCTCCCCAG
Ucp3	TACCCAACCTTGGCTAGACG	GTCCGAGGAGAGAGCTTGC
Glut4	GACGGACACTCCATCTGTTG	GCCACGATGGAGACATAGC
Myh7	CGCATCAAGGAGCTCACC	CTGCAGCCGCAGTAGGTT
Troponin 1	GTGCCTGGAACATCCCTAAT	TGAGAGGCTGTTCTCTCTGC
Ppar γ	CAGCAACCATTGGGTCAGCTC	CCAGAGCATGGTGCCTTCGCT

β-actin	TAAAGACCTCTATGCCAACACAGT	CACGATGGAGGGGCCGGACTCATC
